# Exploring the Use of Oral Pre-exposure Prophylaxis (PrEP) Among Women from Durban, South Africa as Part of the HIV Prevention Package in a Clinical Trial

**DOI:** 10.1007/s10461-020-03072-0

**Published:** 2020-10-26

**Authors:** Ivana Beesham, Renee Heffron, Shannon Evans, Jared M. Baeten, Jenni Smit, Mags Beksinska, Leila E. Mansoor

**Affiliations:** 1 MatCH Research Unit (MRU), Department of Obstetrics and Gynaecology, Faculty of Health Sciences, University of the Witwatersrand, Durban, South Africa; 2 Department of Global Health, Department of Epidemiology, University of Washington, Seattle, WA USA; 3 Department of Global Health, Department of Epidemiology, Department of Medicine, University of Washington, Seattle, WA USA; 4 Centre for AIDS Programme of Research in South Africa (CAPRISA), University of KwaZulu-Natal, Durban, South Africa

**Keywords:** Oral pre-exposure prophylaxis, Clinical trials, HIV prevention, Women

## Abstract

HIV endpoint-driven clinical trials in Africa enroll women who are at heightened risk of acquiring HIV. In 2017, the South African Medical Research Council recommended the provision of oral pre-exposure prophylaxis (PrEP) in HIV prevention trials, at which time the Evidence for Contraceptive Options and HIV Outcomes trial was ongoing and began to provide PrEP on-site at some trial sites. We interviewed 132 women who initiated PrEP on-site at the Durban, South Africa trial site to explore PrEP use, and conducted phone-based interviews 4–6 months post-trial exit to explore post-trial PrEP access. PrEP uptake was high (42.6%). Among women initiating PrEP on-site, 87.9% felt at risk of acquiring HIV. Most women (> 90%) heard of PrEP for the first time from study staff and three-quarters who initiated PrEP on-site continued at trial-exit. PrEP use declined post-trial exit with more than 50% of women discontinuing PrEP, and barriers relating to access emerged.

## Introduction

South Africa (SA) has the largest HIV epidemic in the world [1]. In 2018, there were 7.7 million people living with HIV and 240,000 new infections, with women more disproportionately affected than men, and more than double the number of new infections occurring in young women (15–24 years) compared to men in the same age group [2]. Increased HIV prevention efforts are needed to combat the HIV epidemic. In 2015, the World Health Organization (WHO) made the recommendation that daily oral pre-exposure prophylaxis (PrEP) containing tenofovir disoproxil fumarate (TDF) should be used as a prevention choice for people at substantial risk of HIV infection as part of combination prevention approaches [3]. SA began its PrEP rollout to select sex worker sites in 2016, and later expanded to include additional key populations, including men who have sex with men (MSM), serodiscordant couples, and adolescent girls and young women [4]. Recently, since 2018, access to PrEP in SA has expanded and PrEP is currently available in public sector health facilities within the country [5], in addition to demonstration projects and observational studies [6].

In the context of clinical trials, ethical guidelines recommend that appropriate counselling and access to “state-of-the-art” HIV risk reduction methods are provided to participants in biomedical HIV prevention trials [7]. In SA, the national policy on PrEP which was released in 2016, recommends that the provision of PrEP be framed in a human-rights-based approach ensuring the rights to dignity, non-discrimination, privacy, confidentiality, and the right to services are upheld [8]. The HIV Prevention Trials Network (HPTN) guidance states that in partnership with key stakeholders, HPTN should establish a package of effective, comprehensive and locally sustainable prevention services to be offered to participants [9]. In November 2017, the South African Medical Research Council (SAMRC) recognised the limited availability of PrEP in SA and recommended the provision of PrEP in HIV prevention trials, along with the support of ethical committees, and community consultation and involvement [10]. Biomedical prevention trials have taken different approaches regarding PrEP that have included the provision of information only, the provision of information and referral for PrEP, and on-site provision of PrEP [11].

In this study, we collected data on PrEP use among women who initiated PrEP on-site as part of the HIV prevention package during the Evidence for Contraceptive Options and HIV Outcomes (ECHO) trial. The ECHO trial was conducted between 2015 and 2018, and enrolled 7830 HIV negative women, aged 16 to 35 years, from 12 sites in four African countries [12]. Women desiring contraception were randomized to intramuscular depot medroxyprogesterone acetate (DMPA-IM), a copper intrauterine device (IUD), or a levonorgestrel (LNG) implant, and followed for 12–18 months. The primary ECHO trial outcome was HIV incidence, which was 4.5 per 100 woman-years at the SA trial sites [13]. During the ECHO trial, all women were counselled about PrEP and those who were interested in PrEP were referred to off-site facilities such as demonstration projects where available, to access PrEP. The ECHO trial was ongoing at the time of the SAMRC recommendation and shifted from offering PrEP via referral to off-site facilities where available, to on-site provision at the SA study sites, in addition to referral. The integration of PrEP delivery into the ECHO trial has been described previously [14]. Here, we present data on the uptake of PrEP, reasons for initiating and discontinuing PrEP, side effects experienced, perceived HIV risk, self-reported adherence to PrEP, disclosure of PrEP use and post-trial access to PrEP among women at one study site in Durban, SA.

## Methods

This ancillary study was nested within the ECHO trial (ClinicalTrials.gov, NCT02550067) [12]. The HIV prevention package provided to all women during the ECHO trial included HIV risk reduction counselling; HIV counselling and testing; sexually transmitted infection (STI) testing, treatment and partner notification of STIs; condom provision; partner HIV counselling and testing, and referral for antiretroviral therapy (ART) in discordant couples. At the SA trial sites, women were initially referred to off-site facilities such as demonstration sites and public sector facilities providing PrEP where available, and during the latter part of the trial, PrEP was provided on-site from March 2018. For the Durban ECHO trial site, prior to on-site PrEP provision, a link had been established in September 2017 with a non-governmental organization (NGO) located in close proximity to the research site, that was providing PrEP at no cost. Women were actively referred to this site for PrEP, if interested from October 2017. Women who were HIV negative, not pregnant or breastfeeding, and who perceived themselves to be at substantial risk for acquiring HIV were eligible. Following the on-site provision of PrEP from March 2018, all women in study follow-up who wanted to initiate PrEP, had requested to do so on-site. Women who initiated PrEP on-site and desired to continue PrEP at the final study visit were given a 3-month supply of PrEP and referred to NGO’s, demonstration projects and public-sector facilities providing PrEP. In addition, women who were interested in initiating PrEP at the final study visit were referred.

The ECHO trial captured minimal information on PrEP use since PrEP was part of the comprehensive HIV prevention package and not a separate research procedure. To collect additional information on PrEP use, we conducted an ancillary study from April 2018 to April 2019 among women who chose to initiate PrEP on-site at the Durban, SA ECHO trial site. All women who chose to initiate PrEP on-site were invited to participate in a structured interviewer-administered questionnaire, in their language of choice, approximately 3 months after initiating PrEP on-site. The questionnaire, which was completed during study follow-up or at the final study visit, explored PrEP use, including reasons for initiating and discontinuing PrEP, side effects experienced, perceived HIV risk, reported adherence to PrEP and disclosure of PrEP use. Among women initiating PrEP on-site, data were also collected at the final ECHO study visit on whether PrEP was being continued, and reasons for discontinuation. In addition, women initiating PrEP on-site who elected to continue using PrEP at the final study visit, and who consented to telephonic follow-up, were contacted via telephone 4 to 6 months after their trial exit to explore post-trial access and continued use of PrEP. Women were asked questions on whether they were still using PrEP, if they had any problems accessing PrEP after the trial had ended, and if they had discontinued using PrEP, reasons for discontinuation. In addition, we collected limited information from source notes on women who initiated PrEP off-site from 2017. Here, we collected data on the number of women referred, the number who initiated PrEP, and the timing of PrEP initiation. Data were entered onto the REDCap® electronic data capture tools hosted at the University of the Witwatersrand [15] and analysed using Stata version 14 (StataCorp, College Station, USA). Descriptive analyses were conducted for the purpose of this study.

Data that were collected during the ECHO trial such as demographics and behavioral risk factors (using case report forms), and STIs (STI testing was conducted at the enrolment and final study visit using PCR Gene Expert testing on provider-collected endocervical swabs) were also included. In addition, we collected data on the number of women who were referred for off-site PrEP provision, and the proportion of these women who had initiated PrEP. Eligibility for on-site PrEP provision included being HIV negative, not pregnant or breastfeeding (as per local guidelines at the time), with at least one month of follow-up remaining in the trial, and attended a study visit from March 2018.

Additional written informed consent was obtained to conduct the ancillary study questionnaire and the follow-up phone calls. For the phone-based interviews, women were asked to confirm their name and surname, and were asked a study specific question to verify that they were the correct participant in the study. Women were also asked if it was a suitable time, and they were in a suitable place (quiet and confidential) to conduct the interview. The interviewers conducted the interviews in a private room that was conducive to conducting phone-based interviews. This study was approved by the University of Witwatersrand Human Research Ethics Committee (Wits HREC) (Reference 141112). For the ECHO trial, participants completed written informed consent, and approval was obtained from Wits HREC and the FHI360 ethics review board.

## Results

Of the 324 women eligible for PrEP when it became available on-site, 138 (42.6%) initiated PrEP. An additional 17 women had initiated PrEP off-site. In total, 132 (132 of 138, 95.7%) women who initiated PrEP on-site consented to participate in the ancillary study. At baseline, the mean age was 24 years (range 18–35 years) and 82 (62.1%) women were ≤ 24 years. A quarter (33, 25.0%) had *Chlamydia trachomatis* and three (2.3%) had *Nesseria gonorrhoeae* detected at enrolment into the ECHO trial. At the time of the questionnaire, most women (116, 87.9%) reported feeling at risk of acquiring HIV. Of these 116 women, 97 (83.6%) women reported inconsistent condom use, 57 (49.1%) women felt that their partner had other sexual partners and approximately one in five women (24, 20.7%) did not know their partner’s HIV status (Table 1).

**Table 1:** Reasons women provided for feeling at risk of acquiring HIV

Reason(s) for feeling at risk of acquiring HIV^a^ (N = 116)	N (%)
I don’t always use a condom when having sex	97 (83.6)
I think my partner has other sexual partners beside me	57 (49.1)
I don’t know my partner’s HIV status	24 (20.7)
I don’t trust my partner	17 (14.7)
I have been diagnosed or treated for an STI	17 (14.7)
I have more than one sexual partner	5 (4.3)
Non-sexual HIV exposure	4 (3.4)
Fear of condoms breaking during sex	4 (3.4)
My partner’s status is HIV positive	1 (0.9)

^a^Multiple responses allowed

The main reasons that women cited for initiating PrEP (n = 132) were to prevent or protect against HIV (98, 74.2%); and partner-related reasons (50, 37.9%) such as distrust of the partner, the partner being unfaithful previously and having a partner whose HIV status was unknown (Table 2). Almost a quarter (28, 21.2%) of the women initiated PrEP due to inconsistent or no condom use.

**Table 2:** Reasons for initiating PrEP

Reason(s) for initiating PrEP^a^ (N = 132)	N (%)
To prevent HIV or protect against HIV/feel “safe”/felt at risk for acquiring HIV	98 (74.2)
Partner-related reasons (distrust of partner/partner was unfaithful/partner’s HIV status unknown)	50 (37.9)
Inconsistent or no condom use	28 (21.2)
Fear of acquiring HIV	8 (6.1)
Novelty	8 (6.1)
Fear of condom breaking during sex/previously experienced broken condom during sex	6 (4.5)
Other people in my family have HIV	3 (2.3)
Fear of being raped/previously raped	3 (2.3)
Method “I could use myself”	2 (1.5)
Having sex under the influence alcohol	2 (1.5)
Other^b^	3 (2.3)

^a^Multiple responses allowed

^b^Other reasons for initiating PrEP: counselled by staff and thought it was a good idea to use, trusts the site to give good advice so decided to use PrEP, has multiple partners and does not use condoms with all of them

Over a third of women (n = 53, 40.2%) reported experiencing side effects since starting PrEP, but only four of the 53 (7.5%) women had ongoing side effects at the time of the questionnaire. Common side effects perceived to be related to PrEP (n = 53) included nausea or vomiting (26, 49.1%), headaches (17, 32.1%), and drowsiness or dizziness (9, 17.0%). The four (7.5%) women who had ongoing side effects reported increased appetite and weight gain; headache, nausea and vomiting; nausea and bad dreams; and vomiting.

Most women had heard about PrEP for the first time from study staff (n = 120, 90.9%). Among the 12 (9.1%) women who had heard about PrEP previously, four (33.3%) heard of PrEP from a public health facility, two (16.7%) from friends, two (16.7%) from the internet, and five (41.7%) from other sources (a poster at a clinic, at an HIV counselling and testing course, from a teacher at high school, from other study participants and at a research event). Most women had disclosed PrEP use (n = 119, 90.2%) including about two-thirds who disclosed to a family member (81, 68.1%), just over a third to their partner(s) (46, 38.7%), and just over a third to a friend (44, 37.0%).

**Fig. 1:**
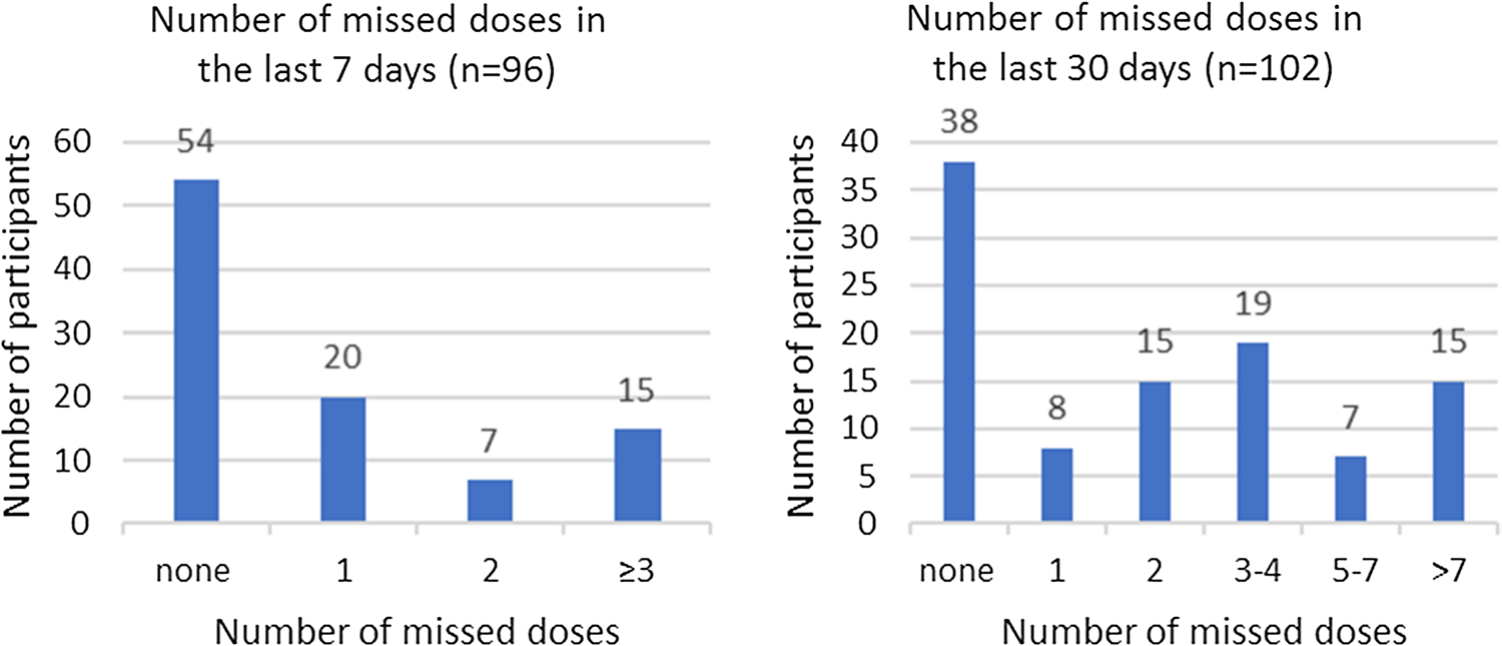
Missing doses in the last 7 and last 30 days

Self-reported adherence to PrEP was collected over the prior seven, and prior 30 days at the time of the questionnaire. About half of the women (54 of 96, 56.3%) reported no missed doses within the past 7 days, and about one-third (38 of 102, 37.3%) reported no missed doses in the last 30 days (Fig. 1). The main reasons for missing doses in the last 30 days (n = 64) were being away from home when tablets needed to be taken (33, 51.6%), and forgetful to take tablets (33, 51.6%) (Table 3).

**Table 3:** Reasons for missed doses in the last 30 days

Reason(s) for missed doses^a^ (N = 64)^b^	N (%)
I was away from home when I needed to take my tablets	33 (51.6)
I forgot to take my tablets	33 (51.6)
I did not have time	8 (12.5)
Alcohol related	5 (7.8)
I ran out of tablets	4 (6.3)
I was having side effects	3 (4.7)
Work related	3 (4.7)
Unable to return to site for a refill	3 (4.7)
I needed a break	2 (3.1)
I did not feel I was at risk anymore	2 (3.1)
Being away from home for long periods and did not take tablets with	2 (3.1)

^a^Multiple responses allowed

^b^102 women were using PrEP in the last 30 days, 38 of these reported none missed doses. Reasons for missed doses among the remaining 64 women are presented

In total, 100 (75.8%) women had decided to continue PrEP at the ECHO study exit visit and 32 (24.2%) women had discontinued PrEP either during study follow-up or at the final study visit. Of the 32 women who discontinued PrEP, 12 (37.5%) stopped due to side effects, five (15.6%) due to partner or family influence, four (12.5%) due to forgetfulness to take PrEP, two (6.3%) were scared of potential side effects and three (9.4%) were unable to return to the research site for a refill. Side effects (n = 12) that led to PrEP being discontinued were nausea or vomiting (7, 58.3%), increased appetite (3, 25.0%), fatigue (3, 25.0%), headache (2, 16.7%), weight gain (1, 8.3%), weight loss (1, 8.3%), dizziness (1, 8.3%), diarrhoea (1, 8.3%), sweating (1, 8.3%) and itchiness of the skin (1, 8.3%).

The following reasons were provided for discontinuing PrEP due to partner or family influence. One participant reported her mother found her PrEP pills and had lots of questions and said to her, “*Are you sure they are not giving you the pills to make you get HIV easily? Ever since I was born I never heard of such a thing*.” A second participant reported that her mother was not happy about her taking PrEP and told her that she should stop. The participant said, “*I don’t think she understands these things*.” A third participant reported her partner said that there was no need for her to take PrEP and disposed of her tablets. A fourth reported she told her mother about PrEP upon initiating PrEP, but her mother was still unable to understand even after she explained, and her mother said, “*these stupid pills*” of yours and that the participant should not take the tablets. Finally, a fifth participant reported that her mother felt she is too young to be taking pills.

Of the 100 women continuing PrEP at study exit, 87 (87.0%) women were contacted telephonically 4 to 6 months post study exit and 41 (47.1%) of these women reported ongoing PrEP use. Among the 41 women, 13 (31.7%) women accessed PrEP from a facility (public sector or NGO) providing PrEP and the remaining 28 (68.3%) reported having leftover tablets from the ECHO trial. Reasons for discontinuing PrEP (n = 46) after the study had ended included “not knowing” or being unable to find a facility providing PrEP (11, 23.9%), feeling that the facility was located too far away from the participants home (7, 15.2%), and not having enough money for transport to get to a facility providing PrEP (6, 13.0%) (Table 4).

**Table 4:** Reasons for discontinuing PrEP post study exit

Reason(s) for discontinuing PrEP^a^ (N = 46)	N (%)
Did not know the facility providing PrEP/unable to find the facility providing PrEP	11 (23.9)
Facility providing PrEP was too far away	7 (15.2)
Did not have the time to go to a facility to access PrEP	7 (15.2)
Lost referral letter	6 (13.0)
Did not have enough transport money to get to facility providing PrEP	6 (13.0)
Relocated out of Durban	5 (10.9)
No longer felt at risk for acquiring HIV	4 (8.7)
Unable to go because of work	4 (8.7)
Went to another facility that did not provide PrEP	2 (4.3)
Stopped because “got tired” of taking tablets	2 (4.3)
Other^b^	6 (13.0)

^a^Multiple responses allowed

^b^Other reasons for discontinuing PrEP post study exit: joined another study, was hospitalised, partner wanted me to stop, it was the festive season and I was forgetful to take tablets, I was temporarily away from home for a funeral, and I didn’t feel I needed the tablets

A total of 61 women were referred off-site to access PrEP prior to the on-site availability of PrEP provision. Of these, about a quarter (17, 27.9%) were started on PrEP at the off-site facility. Eighteen (29.5%) of these women who were referred for PrEP had not initiated PrEP off-site but returned to the study site to initiate PrEP on-site when it became available.

## Discussion

To our knowledge, the ECHO trial was one of the first trials to provide PrEP on-site as part of the HIV prevention package [12]. We observed a high uptake of PrEP at the Durban, SA site, with almost one in two eligible women initiating PrEP, and nearly all on-site. Women who initiated PrEP felt that they were at high risk of acquiring HIV. Surprisingly, most women (> 90%) had heard about PrEP for the first time from trial staff. Both the use of PrEP and access to PrEP had declined post-trial exit.

High rates of PrEP uptake have been seen in other studies among African women where PrEP has been integrated into youth-friendly clinics, family planning clinics and mobile clinics [16]. While PrEP uptake in the FEM-PrEP and VOICE trials was low (20–30%) [17, 18], the uptake of PrEP in HPTN 082 was 95% [19]. However, these studies were designed to study PrEP, while in the ECHO trial, PrEP was offered as part of the HIV prevention package and not specifically as a research question. On the other hand, in HVTN 702, where PrEP was provided as part of HIV standard of prevention during the trial, PrEP use (monitored by self-report and laboratory testing) was low, with only 0.9% of participants reporting PrEP use, and detectable TFV-DP found in only 2.2% of samples [20]. In a “real-world” implementation program in family planning clinics in Kenya, the uptake of PrEP was 22% [21]. Almost 90% of women felt at risk of acquiring HIV and half of these felt their partner had other sexual partners in our study. In HPTN 082, approximately 30% felt their partner had other sexual partners, and both in our study and HPTN 082, over 20% of women did not know their partners HIV status and inconsistent condom use was high [19]. Similar rates of baseline STI infections (*Chlamydia trachomatis* and *Nesseria gonorrhoeae*) were observed in HVTN 702 [20].

In our study, 90% of women had disclosed PrEP use to family members, partners and friends. This contrasts with HPTN 082 where only about 40% of women planned to disclose PrEP use [19]. Stigma has been associated with having a negative influence on both the disclosure of PrEP use and continuation [22]. Five women in our study discontinued PrEP due to the influence of the partner or their mother. This is an important consideration in the planning and execution of clinical trials providing PrEP, and PrEP delivery programs, and highlights the need for broader community education and engagement. In the context of clinical trials, clinicians, counsellors and other study staff should be aware of these factors and be able to employ strategies that can assist participants with disclosure of PrEP use where needed. Community outreach teams and advisory boards can play a role in community education and awareness of HIV prevention methods available, including PrEP. Study staff were found to be vital in the delivery of PrEP in the ECHO trial, and we found that > 90% of participants had heard about PrEP for the first time from study staff. PrEP awareness and demand creation are essential components of PrEP provision [16]. In contrast, a study done in SA primarily among female sex workers and MSM found that only a third of participants had never heard of PrEP [23].

An important consideration in PrEP provision in clinical trials is the issue of post-trial access. The SAMRC noted that post-trial PrEP access could not be supported due to limited funding [10]. In the ECHO trial, we provided a 3-month PrEP supply at exit and other trials providing PrEP should similarly plan to bridge women to publicly available PrEP programs by providing a supply of PrEP at study exit. We found less than half of the women reported ongoing PrEP use after exiting the ECHO trial, and only a third of these had accessed a facility providing PrEP. Of concern is that many women reported having “leftover” tablets from the ECHO trial implying that PrEP adherence might wane over time, or that PrEP use might be intermittent, and this is an area that needs exploration in future research studies. In HPTN 082, detectable plasma tenofovir dropped from 65% at month 3, to 47% at month 6, and only 25% at month 12 [19]. Of the women that discontinued PrEP post-ECHO trial exit, PrEP continuation was limited by structural factors such as poverty and access. A contributing factor could also be the changes experienced by women in accessing PrEP in a trial setting compared to an NGO or public facility. These structural factors are crucial as large-scale rollout of PrEP is considered in SA, and facilities should be located close to where potential clients reside thus reducing barriers like distance and transport costs. The integration of PrEP into existing community facilities e.g. family planning clinics might also eliminate some barriers to access. Furthermore, the number of public sector facilities providing PrEP in SA has recently expanded, thereby increasing access.

Several women reported missing doses of PrEP with more than 50% of participants reporting missing ≥ 2 doses over the prior 30 days, implying protection might have been inadequate as at least 6 doses a week are required for adequate protection in non-rectal HIV exposure [24]. Reasons for missing doses because participants forgot, were busy, and feared or experienced side effects have been documented in other studies [25]. One strategy to foster PrEP adherence and persistence has been to integrate PrEP refills with other reproductive health services e.g. contraception [16]. In the ECHO trial, PrEP follow-up visits were aligned with study follow-up visits where possible. This might have contributed to more women continuing PrEP for the duration of the study. Reasons for stopping PrEP in other studies have included low perceived HIV risk, access, side effects, not wanting to take a daily pill and stigma [26] which were similar to what we found in our study. Overall, adherence (as measured by plasma TFV) has varied in other studies ranging from 24% in FEM-PrEP to 81% in the Partners PrEP Study [27].

This study has some limitations. PrEP was introduced relatively late in the trial, therefore, the maximum follow-up time each woman had with on-site PrEP use was limited to approximately 6 months and some data, e.g. persistence on PrEP is limited by follow-up time. We used a structured questionnaire which was administered at one study site in Durban, therefore our findings might not be applicable to different geographical settings and sites. Additional in-depth information is needed to better understand dynamics of PrEP use, including post-trial access. Objective markers of adherence would have been useful to enhance our understanding of adherence as well as greater data on the impact of PrEP use for the women in the trial. However, our study has several strengths. The ECHO trial was one of the first studies to successfully provide PrEP on-site and this is one of the first studies to explore the voluntary use of PrEP by interviewing women who initiated PrEP on-site. There was a high uptake of PrEP. Reasons for starting and stopping PrEP can be of use to other clinical trials providing PrEP, as well as PrEP implementation programs. It is well documented that participants might over-report adherence, however in our study we found that many women reported missing doses and provided reasons that could be useful for PrEP implementation programs and adherence counselling.

## Conclusions

Our study findings strongly support offering PrEP on-site as part of HIV prevention in clinical trials with HIV endpoints. We found that there was a high uptake of PrEP by women who perceived themselves to be at high risk for HIV acquisition and study staff were vital in increasing awareness of, and delivering PrEP. However, we found that > 50% of women discontinued PrEP after exiting the study with barriers relating to PrEP access emerging.
